# Model fit evaluation in multilevel structural equation models

**DOI:** 10.3389/fpsyg.2014.00081

**Published:** 2014-02-05

**Authors:** Ehri Ryu

**Affiliations:** Department of Psychology, Boston CollegeChestnut Hill, MA, USA

**Keywords:** multilevel structural equation model, model fit, fit indices, model fit statistics, level-specific fit evaluation

## Abstract

Assessing goodness of model fit is one of the key questions in structural equation modeling (SEM). Goodness of fit is the extent to which the hypothesized model reproduces the multivariate structure underlying the set of variables. During the earlier development of multilevel structural equation models, the “standard” approach was to evaluate the goodness of fit for the entire model across all levels simultaneously. The model fit statistics produced by the standard approach have a potential problem in detecting lack of fit in the higher-level model for which the effective sample size is much smaller. Also when the standard approach results in poor model fit, it is not clear at which level the model does not fit well. This article reviews two alternative approaches that have been proposed to overcome the limitations of the standard approach. One is a two-step procedure which first produces estimates of saturated covariance matrices at each level and then performs single-level analysis at each level with the estimated covariance matrices as input (Yuan and Bentler, 2007). The other level-specific approach utilizes partially saturated models to obtain test statistics and fit indices for each level separately (Ryu and West, 2009). Simulation studies (e.g., Yuan and Bentler, 2007; Ryu and West, 2009) have consistently shown that both alternative approaches performed well in detecting lack of fit at any level, whereas the standard approach failed to detect lack of fit at the higher level. It is recommended that the alternative approaches are used to assess the model fit in multilevel structural equation model. Advantages and disadvantages of the two alternative approaches are discussed. The alternative approaches are demonstrated in an empirical example.

## Introduction

Research in the behavioral sciences increasingly employs multilevel multivariate research designs. In multilevel data, also called hierarchically structured or clustered data, lower-order units are clustered within higher-order units, for example, students within classrooms. Multilevel data structures may also arise in studies involving repeated measurements (e.g., diary data) collected from multiple individuals. In analyzing multilevel data, the need for multilevel modeling is twofold. First, observations taken from the lower-order units typically do not meet the independence assumption, because observations in the same cluster are likely to be more homogenous than those from different clusters. When the dependency due to clustering is ignored, the analysis yields incorrect standard errors and therefore invalid statistical inferences. Multilevel modeling takes this dependency into account and provides appropriate adjustment for the standard errors leading to valid statistical inferences. Second, in multilevel research, the relationship between variables at one level does not necessarily generalize to the relationship between the same set of variables at another level. Multilevel modeling allows researchers to investigate the relationships between variables at different levels in the hierarchical structure.

Structural equation modeling (SEM, Jöreskog, 1978; Bentler, 1980) is a general framework for modeling and analyzing multivariate data. In SEM, latent variable models can be specified to estimate the relationships between latent constructs and observed indicators, and a set of linear relationships with more than one dependent variable can be estimated simultaneously. Multilevel structural equation modeling (MSEM) is a general framework which combines SEM and multilevel modeling simultaneously. In other words, MSEM is an advanced SEM technique developed for multilevel research. MSEM can be applied to a wide range of applications that involve multilevel multivariate data, including multilevel factor analysis and multilevel path analysis models. With the theoretical development of MSEM (e.g., Goldstein and McDonald, 1988; Muthén, 1990, 1994; Longford and Muthén, 1992; Muthén and Satorra, 1995) together with the development of software packages, the use of MSEM in behavioral and social science research is becoming increasingly popular.

Like other applications of SEM, applications of MSEM usually address two issues: (a) assessing the goodness of fit of the hypothesized model to the data, and (b) estimating and testing individual parameters in the hypothesized model. Issue (a) assesses how well a hypothesized model approximates the multivariate structure underlying the set of variables. Issue (b) can only be addressed in a meaningful way in a well-fitting model. A poor fitting model does not approximate the underlying structure observed in the data; the parameter estimates obtained in the poor fitting model cannot be interpreted as reasonably summarizing the relationships between the variables (Browne and Cudeck, 1993; West et al., 2012).

In MSEM, it has been a standard approach that the model fit is evaluated using test statistics and fit indices that were developed for model fit evaluation in single-level SEM. In the standard approach, the entire multilevel model is evaluated simultaneously. In multilevel data, effective sample size is typically much larger at the lower level than at the higher level. The model fit evaluation obtained by the standard approach is likely to be dominated by the model fit at the lower level, and may not be sensitive to detect lack of fit at the higher level (Yuan and Bentler, 2007; Ryu and West, 2009; Hox, 2010). Also when the standard approach results in poor model fit, it does not indicate whether the model fit is poor at the lower level, at the higher level, or at both levels.

Two alternative approaches have been proposed to the evaluation of model fit in MSEM. This article reviews the standard approach followed by a discussion of its limitations. Then the alternative approaches are introduced. Model fit evaluation methods are illustrated using an empirical data set. Practical recommendations are provided for researchers who wish to adopt MSEM in their research.

## Multilevel structural equation model

Throughout this article, I use *individual* to indicate level-1 unit and *cluster* to indicate level-2 unit. Suppose that data are collected from *N* individuals (*i* = 1, 2, …, *N*) nested within *J* (*j* = 1, 2, …, *J*) clusters. The clusters are a simple random sample from a population of clusters, and the individuals are a simple random sample within each cluster. At level 2, the observations taken from clusters are independent of one another. At level 1, the individual-level observations are independent of one another within each cluster, but not across clusters.

Let ***y***_*ij*_ denote a data vector for individual *i* in cluster *j*. In multilevel data, there are two sources of random variation: random variation due to between-cluster differences at level 2 and random variation due to between-individual differences within clusters at level 1. In MSEM, the data vector is decomposed into means, between-cluster random components, and within-cluster random components as shown in Equation (1):

(1) $$y_{ij}=\mu +y_{Bj}+y_{Wij}$$

where $E\left(y_{Bj}\right)=E\left(y_{Wij}\right)=0$. Note that ***y***_*Bj*_ and ***y***_*Wij*_ are latent (i.e., not directly observed) random components that reflect between-cluster and within-cluster variation, respectively. In MSEM, all level-1 variables are subject to the model-based decomposition shown in Equation (1). For level-2 variables, the decomposition can be simplified because the level-1 random components are zero (i.e., ***y***_*Wij*_ = 0).

Based on the decomposition shown in Equation (1), the mean and covariance structure of ***y***_*ij*_ are:

(2) $$E\left(y_{ij}\right)=\mu$$

(3) $$\text{Cov}\left(y_{ij}\right)=\text{Cov}\left(y_{Bj}\right)+\text{Cov}\left(y_{Wij}\right)\text{ or }\Sigma_{y}=\Sigma_{B}+\Sigma_{W}$$

The covariance structure of ***y***_*ij*_ is decomposed into level-1 and level-2 covariance structures as shown in (3) based on two assumptions. First, the level-1 and level-2 random components are uncorrelated [i.e., $\text{Cov}\left(y_{Bj},y_{Wij}\right)=0$]. Second, the level-1 covariance structure is homogeneous across clusters (i.e., **Σ**_*Wj*_ = **Σ**_*W*_ for all *j*). A typical MSEM model consists of the level-2 covariance structure **Σ**_*B*_ (**θ**) and level-1 covariance structure **Σ**_*W*_ (**θ**), and mean structure *μ* (**θ**), where **θ** is a vector of parameters in the hypothesized model. In many applications, the mean structure is specified as a saturated model (i.e., the number of parameters in the mean structure is equal to the number of means in the data).

Assuming multivariate normality for each of the level-1 and level-2 random components, the maximum likelihood *(ML)* solution is obtained using the fitting function in Equation (4) (Bentler and Liang, 2003; Liang and Bentler, 2004).

(4) $$\begin{array}{l} F_{ML}=\sum_{j\;=\;1}^{J}\left[\log\left|\Sigma_{gj}\left(\theta \right)\right|+tr\left(\Sigma_{gj}^{-1}\left(\theta \right)S_{Bj}\right)\right]\\ \;+\sum_{j\;=\;1}^{J}\left(n_{j}-1\right)\left[\log\left|\Sigma_{W}\left(\theta \right)\right|+tr\left(\Sigma_{W}^{-1}\left(\theta \right)S_{Wj}\right)\right] \end{array}$$

where *n*_*j*_ = number of individuals in group *j*, $S_{Bj}=n_{j}\left({\bar{y}}_{j}-\bar{y}\right){\left({\bar{y}}_{j}-\bar{y}\right)}^{\prime }$ in which ${\bar{y}}_{j}=n_{j}^{-1}\sum_{i=1}^{n_{j}}y_{ij}$ and $\bar{y}=N^{-1}\sum_{j=1}^{J}\sum_{i=1}^{n_{j}}y_{ij}$, $S_{Wj}={\left(n_{j}-1\right)}^{-1}\left(y_{ij}-{\bar{y}}_{j}\right){\left(y_{ij}-{\bar{y}}_{j}\right)}^{\prime }$, and **Σ**_*gj*_ (**θ**) = **Σ**_*W*_ (**θ**) + *n*_*j*_ **Σ**_*B*_ (**θ**). For perfectly balanced case in which *n*_*j*_ = *n* for all *j*, the *ML* fitting function simplifies to

(5) $$\begin{array}{l} F_{ML}=J\left[\log\left|\Sigma_{gj}\left(\theta \right)\right|+tr\left(\Sigma_{gj}^{-1}\left(\theta \right)S_{Bj}\right)\right]\\ \;+\left(N-J\right)\left[\log\left|\Sigma_{W}\left(\theta \right)\right|+tr\left(\Sigma_{W}^{-1}\left(\theta \right)S_{Wj}\right)\right] \end{array}$$

The first term of the *ML* fitting function reflects the lack of fit in the level-2 covariance structure, and the second term reflects the lack of fit in the level-1 covariance structure. Note that the first and second terms are differentially weighted by the level-2 sample size *J* and the effective level-1 sample size (*N* − *J*).

## “Standard” model fit evaluation

This section briefly reviews the test of exact fit and fit indices (CFI and RMSEA) produced by the “standard” approach to evaluating model fit in MSEM. The standard approach refers to the conventional method in which the goodness of fit is examined for the entire multilevel structural equation model simultaneously. Note that the standard approach parallels to the model fit evaluation in single-level SEM.

### Test of exact fit

The standard test of exact fit test the joint hypothesis *H*_0_: **Σ**_*B*_ = **Σ**_*B*_ (**θ**) and **Σ**_*W*_ = **Σ**_*W*_ (**θ**). **Σ**_*B*_ and **Σ**_*W*_ are level-2 and level-1 covariance structures that underlie *y*_*Bj*_ and *y*_*Wij*_ in the population. **Σ**_*B*_ (**θ**) and **Σ**_*W*_ (**θ**) are the model-implied level-2 and level-1 covariance structures, respectively, that are reproduced by the hypothesized model with parameters **θ**. The test of exact fit is obtained using the likelihood ratio test between the saturated model [i.e., just identified model with degrees of freedom (*df*) = 0] and the hypothesized model with a positive *df*. The ML test statistic *T*_*ML*_ is obtained by

(6) $$T_{ML}=F_{ML}\left(\hat{\theta }\right)-F_{ML}\left({\hat{\theta }}_{S}\right)$$

where $F_{ML}\left(\hat{\theta }\right)$ is the ML fitting function value for the hypothesized model and $F_{ML}\left({\hat{\theta }}_{S}\right)$ is the ML fitting function value for the saturated model. Under the assumptions of proper model specification, multivariate normality, and sufficiently large *J, T*_*ML*_ follows a chi-square distribution. The *df* is equal to the difference in the number of parameters between the hypothesized and the saturated models. If the model-implied covariance structure fits the unrestricted covariance structure exactly in the population, *T*_*ML*_ follows a central chi-square distribution with *df* = difference in the number of parameters between the hypothesized and the saturated models. If exact fit does not hold in the population, *T*_*ML*_ follows a non-central chi-square distribution with the same *df* and non-centrality parameter λ = (*N* − 1)*F*_0_, where *F*_0_ is the ML fitting function value reflecting lack of fit in the population (MacCallum et al., 1996).

### Comparative fit index (CFI)

The CFI (Bentler, 1990) is a fit index that measures goodness of fit of the hypothesized model compared to a baseline model. Typically an independence model in which the variances are estimated freely without any constraints and all the covariances are fixed to zero is used as the baseline model^1^. In Equation (7), Δ compares the non-centrality parameter in the hypothesized model to the non-centrality parameter in the baseline model.

(7) $$\Delta =1-\frac{\lambda_{\text{Hypothesized}}}{\lambda_{\text{Baseline}}}$$

When the fit of the hypothesized model is as poor as the fit of the baseline model, Δ becomes zero. The better the fit of the hypothesized model than the fit of the baseline model, the closer Δ approaches to 1. Equivalently, Δ can be obtained using the chi-square test statistic and the *df*.

(8) $$\text{CFI}=1-\frac{\text{Max}\left[\left(\chi_{\text{Hypothesized}}^{2}-df_{\text{Hypothesized}}\right),0\right]}{\text{Max}\left[\left(\chi_{\text{Baseline}}^{2}-df_{\text{Baseline}}\right),0\right]}$$

### Root mean squared error of approximation (RMSEA)

The RMSEA (Steiger, 1990; Browne and Cudeck, 1993) provides a measure of lack of fit in the population with an adjustment for the parsimony of the model. RMSEA attempts to estimate the error of approximation of the model in the population apart from the error of estimation due to sampling error. The sample ML fitting function value is a biased estimator of the population fitting function value. A less biased estimator is obtained by Equation (9) (MacCallum et al., 1996).

(9) $${\hat{F}}_{0}={\hat{F}}_{ML}-\frac{df}{\left(N-1\right)}$$

RMSEA is a measure of lack of fit in the population per degree of freedom. From Equation (9), RMSEA is obtained by

(10) $$\text{RMSEA}=\sqrt{\frac{{\hat{F}}_{0}}{df}}=\sqrt{\text{Max}\left[\left(\frac{\chi^{2}-df}{df\left(N-1\right)}\right),0\right]}$$

## Limitations of the standard approach

In the standard approach, the test statistic for exact fit test is obtained using the ML fitting function shown in Equations (4) and (5), in which the lack of fit at level 1 and the lack of fit at level 2 are weighted differentially by (*N* − *J*) and *J*, respectively. The fit indices CFI and RMSEA are calculated using the chi-square test statistic, and the goodness of fit or the lack of fit at each level is not equally reflected in these fit indices. Therefore the model fit evaluation obtained by the standard approach is likely to be dominated by the level-1 model, and may not be sensitive to detect lack of fit in the level-2 model (Yuan and Bentler, 2007; Ryu and West, 2009; Hox, 2010). Also when the standard approach results in poor model fit, it does not indicate whether the model fit is poor at level 1, at level 2, or at both levels.

Two alternative approaches have been proposed to overcome the limitations of the standard approach. Both approaches assess the model fit at each level separately to overcome the limitations of the standard approach. The *level-specific model fit evaluation* by Ryu and West (2009) utilizes partially saturated models to evaluate the fit of the level-1 and level-2 models separately. The *segregating procedure* by Yuan and Bentler (2007) separates multilevel covariance structure into multiple single-level covariance structure models.

## Level-specific model fit evaluation

In the literature, the idea of using partially saturated models for separate assessment of model fit at each level was initially suggested by Hox (2002, 2010). Based on this idea, Ryu and West (2009) developed level-specific test statistics for exact fit test, CFIs, and RMSEAs, and investigated the performance of the level-specific statistics in a simulation study. In this approach, a partially saturated model in which the level-1 model is saturated (i.e., just identified with *df* = 0) is used to assess the model fit at level 2; a partially saturated model in which the level-2 model is saturated is used to assess the model fit at level 1. In this article, subscripts PS_B and PS_W are used to denote level-specific model fit statistics obtained using the partially saturated models. The subscript PS_B indicates that the statistics evaluates the goodness of fit at level 2. The subscript PS_W indicates that the statistic evaluates the fit at level 1.

### Level-specific test of exact fit

For assessing model fit at level 2, a partially saturated model is specified in which the level-1 model is saturated ([**Σ**_*B*_ (**θ**), **Σ**_*W*_ (**θ**;_*S*_)]), where **θ** is a vector of parameters in a hypothesized model and **θ**_*S*_ is a vector of parameters in a saturated model. From Equation (6), the test statistic for this partially saturated model is

(11) $$\begin{array}{l} \chi_{\text{PS}\_\text{B}}^{2}=F_{ML}\left[\Sigma_{B}\left(\hat{\theta }\right),\Sigma_{W}\left({\hat{\theta }}_{S}\right)\right]\\ \;-F_{ML}\left[\Sigma_{B}\left({\hat{\theta }}_{S}\right),\Sigma_{W}\left({\hat{\theta }}_{S}\right)\right] \end{array}$$

Any lack of fit captured in Equation (11) is due to the discrepancy between $\Sigma_{B}\left(\hat{\theta }\right)$ and $\Sigma_{B}\left({\hat{\theta }}_{S}\right)$. Therefore the level-specific test statistic for exact fit at level 2 is obtained by Equation (11) by comparing a partially saturated model [**Σ**_*B*_ (**θ**), **Σ**_*W*_ (**θ**_*S*_)] to a fully saturated model [**Σ**_*B*_ (**θ**_*S*_), **Σ**_*W*_ (**θ**_*S*_)]. The *df* is the difference in number of parameters between the hypothesized and saturated models at level 2.

Another partially saturated model [**Σ**_*B*_ (**θ**_*S*_), **Σ**_*W*_ (**θ**)] is used for assessing model fit at level 1. The level-specific test statistic shown in Equation (12) serves as the test of exact fit in the level-1 model.

(12) $$\begin{array}{l} \chi_{\text{PS}\_\text{W}}^{2}=F_{ML}\left[\Sigma_{B}\left({\hat{\theta }}_{S}\right),\Sigma_{W}\left(\hat{\theta }\right)\right]\\ \;-F_{ML}\left[\Sigma_{B}\left({\hat{\theta }}_{S}\right),\Sigma_{W}\left({\hat{\theta }}_{S}\right)\right] \end{array}$$

The *df* is the difference in number of parameters between the hypothesized and saturated models at level 1.

### Level-specific CFIs

CFI_PS_B_ and CFI_PS_W_ are computed using the level-specific test statistics shown in Equations (11) and (12), respectively. The baseline models should also be partially saturated. For level-2 model fit evaluation, an appropriate baseline model is a partially saturated independence model in which the level-2 model is an independence model and the level-1 model is a saturated model. CFI_PS_B_ is computed by the same formula as in Equation (8) using the statistics and degrees of freedom obtained from the partially saturated model and the partially saturated independence model. For level-1 model fit evaluation, a partially saturated independence model in which the level-1 model is an independence model and the level-2 model is a saturated model serves as an appropriate baseline model to compute CFI_PS_W_.

### Level-specific RMSEAs

As mentioned earlier, RMSEA is a measure of lack of fit in population per *df*. An unbiased estimator of the level-specific fitting function value in the population would be desirable level-specific RMSEA. The ML fitting function shown in Equation (5) can be rewritten as

(13) $$F_{ML}=\left(J\right)F_{\text{ML}\_\text{B}}+\left(N-J\right)F_{\text{ML}\_\text{W}}$$

where *F*_ML_B_ captures the discrepancy between model and data in the level-2 covariance structure, and *F*_ML_W_ captures the discrepancy in the level-1 covariance structure. Using Equations (9) and (10), RMSEA_PS_B_ can be obtained by

(14) $${\text{RMSEA}}_{\text{PS}\_\text{B}}=\sqrt{\text{Max}\left[\left(\frac{\chi_{\text{PS}\_\text{B}}^{2}-df_{\text{PS}\_\text{B}}}{df_{\text{PS}\_\text{B}}\left(J\right)}\right),0\right]}$$

as a less biased estimator of the lack of fit at level 2 in population. Likewise, RMSEA_PS_W_ can be obtained by

(15) $${\text{RMSEA}}_{\text{PS}\_\text{W}}=\sqrt{\text{Max}\left[\left(\frac{\chi_{\text{PS}\_\text{W}}^{2}-df_{\text{PS}\_\text{W}}}{df_{\text{PS}\_\text{W}}\left(N-J\right)}\right),0\right]}$$

as a less biased estimator of the lack of fit at level 1 in population.

### Performance of level-specific fit evaluation

In a simulation study, Ryu and West have empirically shown that the level-specific method successfully detected misspecification both at level 1 and at level 2, whereas the standard approach failed to detect lack of fit in the level-2 model. The CFI and RMSEA obtained by the standard approach incorrectly indicated good model fit (i.e., CFI greater than 0.95 and RMSEA smaller than 0.05, following the conventional rule of thumb; see West et al., 2012, Table 13.1) when the level-2 model was incorrectly specified. Both CFI and RMSEA were more biased toward incorrectly indicating good model fit (a) when the number of groups was smaller with the group size held constant, and (b) when the number of groups was smaller for a fixed total sample size. These results imply that the standard approach is more likely to miss the lack of fit in the level-2 model when the sample consists of smaller number of level-2 units for the same total sample size (e.g., the problem of the standard approach is more severe when multilevel data are collected from fewer clusters of larger size than from more clusters of smaller size).

The level-specific test statistics obtained by ML estimation rely on the assumption of multivariate normality. The multivariate normality assumption is important for ML estimator to achieve its asymptotic properties. In a simulation study, Ryu (2011) investigated how level-specific ML statistics are influenced by skewness and kurtosis when the assumption of multivariate normality is not met. Under positive skewness and kurtosis, the statistics were larger than the expected value under multivariate normality, and the Type I error rates for the level-specific exact fit tests were higher than the nominal level. Rule of thumb cutoff values for appreciable bias were skewness ≥ 2 and kurtosis ≥ 7 for the random components at each level. These rule of thumb cutoff values were consistent with the recommendation provided by Curran et al. (1996) for the ML test statistic in single-level SEM. Under positive skewness, the upper tails of the distribution of the level-specific ML statistics were thicker than the theoretical chi-square distribution. (i.e., more likely to yield a larger value). Under positive kurtosis, the upper tails of the distribution became longer (i.e., potentially yielding an extremely large value). The level-specific ML test statistics were affected by positive skewness and kurtosis only when the distributional assumption is violated at the corresponding level, but not when the assumption is violated at the other level.

## Segregating procedure

The Yuan and Bentler (2007) segregating procedure involves two steps. In the first step, the ML estimates for unrestricted level-1 (**Σ**_*W*_) and level-2 (**Σ**_*B*_) covariance matrices, and their asymptotic covariance matrices are obtained. The second step uses the estimated covariance matrices $\hat{\Sigma }$_*W*_ and $\hat{\Sigma }$_*B*_ as if they are observed covariance matrices that are used as input data in single-level SEM.

The ML estimates $\hat{\Sigma }$_*W*_ and $\hat{\Sigma }$_*B*_ are obtained by maximizing the log-likelihood function shown in Equation (16), assuming multivariate normality.

(16) $$\begin{array}{l} l=\sum_{j=1}^{J}[c_{j}-\frac{1}{2}\log\left|\Sigma_{gj}\right|-\frac{n_{j}}{2}{\left({\bar{y}}_{j}-\mu \right)}^{\prime }\Sigma_{gj}^{-1}\left({\bar{y}}_{j}-\mu \right)\\ \;-\frac{\left(n_{j}-1\right)}{2}\log\left|\Sigma_{W}\right|-\frac{1}{2}\sum_{i=1}^{n_{j}}{\left(y_{ij}-{\bar{y}}_{j}\right)}^{\prime }\text{​}\Sigma_{W}^{-1}\left(y_{ij}-{\bar{y}}_{j}\right)] \end{array}$$

where **Σ**_*gj*_ = **Σ**_*W*_ + *n*_*j*_ **Σ**_*B*_. Yuan and Bentler (2002, 2005, 2007) theoretically showed an asymptotic distribution in which the ML estimates in MSEM converges. For the segregating procedure, Yuan and Bentler (2007) showed that when the level-2 sample size *J* approaches infinity or the average group size *n* approaches infinity, the distribution of the ML estimates for unrestricted level-1 covariance matrix asymptotically converges in a normal distribution, and the asymptotic covariance matrix equals to the inverse of the normal theory based information matrix that is obtained by fitting the level-1 model in conventional single-level SEM. The distribution of the ML estimates for unrestricted level-2 covariance matrix asymptotically converges in a normal distribution, and the asymptotic covariance matrix equals to the inverse of the normal theory based information matrix that is obtained by fitting the level-2 model in conventional single-level SEM. Based on these asymptotic properties, a multilevel structural equation model can be segregated into two separate single-level structural equation models.

In the second step, the hypothesized level-1 model **Σ**_*W*_ (**θ**) is fit to $\hat{\Sigma }$_*W*_; the hypothesized level-2 model **Σ**_*B*_ (**θ**) is fit to $\hat{\Sigma }$_*B*_. The level-1 and level-2 models are estimated and evaluated separately using single-level SEM. In each model, the model fit is evaluated using the standard procedure (described in section “Standard” Model Fit Evaluation) developed for single-level SEM. Because the level-1 and level-2 models are evaluated separately, the model fit evaluation for the level-2 model is not dominated by the level-1 model.

Yuan and Bentler provided a SAS program for the first step to produce estimates of unrestricted covariance matrices at each level (Multi-Single.sas, freely downloadable from www.nd.edu/~kyuan/multilevel). Their SAS program produces estimates of level-1 and level-2 covariance matrices, but does not produce the mean vector. In many applications of MSEM, the mean structure in the model is specified as a saturated model (i.e., the number of mean parameters is equal to the number of means in the data). In this case, estimating a two-level covariance structure model without a mean structure is not a serious disadvantage of the segregating procedure. For models in which the mean structure is necessary, it is possible to modify the SAS program to produce estimated mean vector, and to use the estimated mean vector and level-2 covariance matrix as input data for the level-2 model with a mean structure.

Yuan and Bentler also proposed five test statistics for evaluating segregated models: maximum likelihood (ML), ML rescaled (MLR), residual-based asymptotically distribution free (RADF), corrected residual-based ADF, (CRADF), and residual-based *F* statistic (*F*_*R*_). Their simulation study showed that each of these statistics was less powerful in detecting poor fit in the level-2 model when applied to the entire multilevel model than when applied to segregated models. The ML statistics resulted in inflated Type I error rates under lognormal distribution. RADF statistics did not perform well even when applied to segregated models under multivariate normality. CRADF and *F*_*R*_ test statistics showed a lower statistical power than the ML and MLR statistics.

The advantage of the segregating procedure over simultaneous evaluation of the entire multilevel model has also been replicated in other studies (Ryu, 2008; Ryu and West, 2009). In these studies, the segregating procedure successfully detected misspecification both at level 1 and level 2, whereas the standard approach failed to detect the lack of fit at level 2. Compared to the level-specific approach, the segregating procedure resulted in a slightly higher non-convergence rate. The model fit evaluation results were comparable for the level-1 model between the level-specific and the segregating approaches. But the two approaches showed small but consistent discrepancies in the model fit statistics for the level-2 model both when the level-2 model was correctly and incorrectly specified. For the level-2 model, the ML test statistics and the RMSEA values were larger in the segregating procedure than those in the level-specific approach.

## Level-specific and segregating approaches: advantages, disadvantages, and practical considerations

In practice, the level-specific approach requires estimating at least two additional multilevel models in order to obtain level-specific model fit evaluation: two partially saturated models. Additional baseline models need to be estimated in order to compute level-specific CFIs. Level-specific test statistics and fit indices are not yet implemented in software packages. Additional computation is needed to obtain level-specific fit statistics. The segregating approach requires two steps: to produce estimated covariance matrices at each level in the first step, and then to estimate the level-1 and level-2 models as separate single-level models using these covariance matrices as input in the second step. Once the first step is completed, the multilevel model is segregated into two single-level models. Model fit is evaluated in two single-level models. Test statistics and fit indices can be obtained using software packages without additional computation.

Yuan and Bentler's SAS program (Multi-Single.sas, www.nd.edu/~kyuan/multilevel) produces estimated level-1 and level-2 covariance matrices and asymptotic covariance matrices for the variance and covariance estimates, for the first step of the segregating procedure. For a set of five variables, for example, the SAS program produces (5 × 5) covariance matrices at each level and (15 × 15) asymptotic covariance matrices for the elements in the (5 × 5) covariance matrices. A limitation of the segregating procedure is that the SAS program may require a huge amount of computer memory and fail to run. This problem may occur when the number of number of variables is large so that the dimension of the covariance matrices and asymptotic covariance matrices will also be large, or when the sample size is large^2^.

It is not recommended that partially saturated models or segregated single-level models are used to obtain parameter estimates and standard errors. The parameter estimates and standard errors should be obtained using MSEM which finds a solution for the set of parameters in the entire hypothesized model. It is possible to use the segregating procedure to produce parameter estimates and standard errors in addition to model fit evaluation. In the past when the availability of SEM software packages with multilevel modeling capability was limited, the segregating procedure allowed researchers to obtain estimates and standard errors for a multilevel structural equation model using single-level technique. However MSEM software packages are widely available now.

Segregating a multilevel model into multiple single-level models introduces potential problems. First, *estimated* covariance matrices are used as input data for model estimation in the second step. The parameter estimates depend on the estimated covariance matrices. In practice, the theoretical properties of the estimated covariance matrices do not necessarily hold with a finite sample size. Second, a multilevel structural equation model is *one* hypothesized model which consists of two components. The estimation method finds a solution which best satisfies the criterion for the set of parameters in the hypothesized model. Change in model specification in one part of the model may influence the estimated solution for parameters in other parts of the model. In the segregating procedure, the level-1 and level-2 models are separated into two models. The estimation method finds a solution to best satisfy the criterion for the set of parameters in the level-1 model, and for the set of parameters in the level-2 model, separately. The solution from separate estimation in multiple single-level models is not necessarily the same as the solution from simultaneous estimation in a multilevel model. Third, research questions often require that constraints are imposed on parameters at different levels, for example, the relationship of math self-confidence with math achievement at the student level is the same as the relationship at the school level. An equality constraint can be easily imposed between the two parameters in MSEM. But in the segregating approach, it is not straightforward to impose a constraint between a parameter in the level-1 model and another parameter in the level-2 model.

## An empirical example

**Figure 1:**
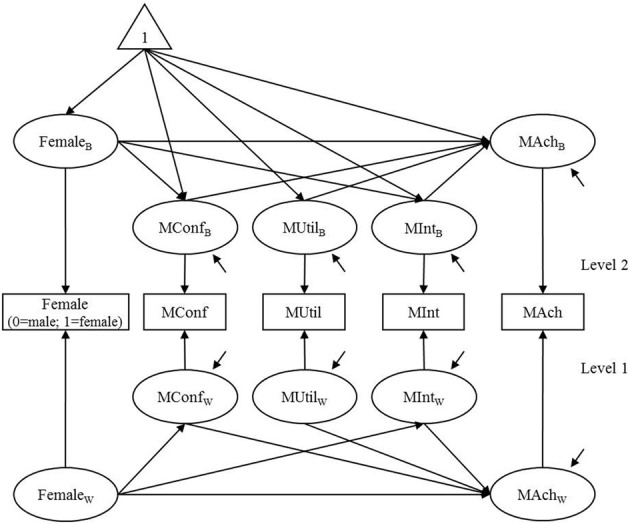
**A two-level path model of mathematics motivation, gender, and achievement**. MConf, self-confidence; MUtil, student's valuing or utility; MInt, student's interest; MAch, mathematics achievement. Female: 1 = female; 0 = male. At level 2, MConf_B_, MUtil_B_, and MInt_B_ are correlated with one another. At level 1, MConf_W_, MUtil_W_, and MInt_W_ are correlated with one another.

This section demonstrates the standard, level-specific, and segregating approaches to evaluating model fit in multilevel structural equation model using an empirical data set from TIMSS (Trends In International Mathematics and Sciences) 2003 International Database (Source: TIMSS 2003 Assessment, Copyright © 2005 International Association for the Evaluation of Educational Achievement; Martin, 2005). The data are from 5928 students clustered in 164 schools in Singapore. The school size ranged from 26 to 42, mean = 36.146, and standard deviation = 2.031. A two-level model shown in Figure 1 depicts hypothesized relationships of mathematics motivation and gender with mathematics achievement (Chiu, 2007). The mathematics motivation variables are self-confidence in mathematics (MConf), student's valuing or utility of mathematics (MUtil), and student's interest in mathematics (Mint). Each of the motivation variables was measured by a composite score of items from a 4-point Likert type scale in which a lower score indicated higher motivation. The Singapore national math Rasch score was used for mathematics achievement (MAch, mean = 150, standard deviation = 10, see TIMSS 2003 User Guide; Martin, 2005).

In the two-level path model, a variable indicating gender of students (Female: 1 = female, 0 = male) is hypothesized to account for each of the motivation variables and achievement. The level-1 variable Female is decomposed into level-1 and level-2 latent random components as shown in Equation (1), as are all the other level-1 variables. Specifically, the level-1 random component Female_W_ is a binary variable distinguishing female and male students; the level-2 random component Female_B_ is a continuous variable indicating proportion of females in each school. At level 1, the relationships of Female_W_ with other mathematics variables represent gender difference among students. At level 2, the relationships of Female_B_ on other mathematics variables represent relationships between the proportion of females and the aggregated level of mathematics variables among schools.

Three models were estimated. I first estimated the model depicted in Figure 1 (Model_C_). Then I estimated two additional models with additional constraints. In Model_MW_, the structural relationship between MConf_W_ and MAch_W_ at level 1 was fixed to zero. In Model_MB_, the structural relationship between Female_B_ and MAch_B_ at level 2 was fixed to zero. Maximum likelihood (ML) estimation in Mplus 7 (Muthén and Muthén, 1998–2012) was used for estimation. For segregating approach, the Yuan and Bentler's SAS program was used to produce estimates of level-1 and level-2 covariance matrices. Then the level-1 and the level-2 models were estimated as separate single-level path models using ML estimation in Mplus 7^3^.

Table 1 presents the model fit statistics obtained by the standard, level-specific, and segregating approaches. All three approaches indicated that Model_C_ fit well both at level 1 and at level 2^4^. Table 2 presents the ML estimates and standard errors for Model_C_^5^. The estimates and standard errors presented under “Multilevel model” were obtained by MSEM. The estimates and standard errors presented under “Segregated model” were obtained by Yuan and Bentler's segregating procedure. The results for the level-1 model were identical (within rounding error) between the multilevel and segregated single-level models. For the level-2 model, the estimates were comparable but the standard errors were smaller in the segregated single-level models than in the multilevel model.

**Table 1:** **Model fit statistics obtained by the standard, level-specific, and segregating approaches**.

	**Model_C_**	**Model_MW_**	**Model_MB_**
**STANDARD APPROACH**			
Test of exact fit	χ^2^ (2) = 1.646, *p* = 0.439	χ^2^ (3) = 257.781^a^	χ^2^ (3) = 12.737, *p* = 0.005
CFI	1.000	0.960	0.998
RMSEA	0.000	0.120	0.023
**LEVEL-SPECIFIC APPROACH FOR LEVEL-2 MODEL (PS_B)**			
Test of exact fit	χ^2^_PS_B_ (1) = 0.895, *p* = 0.344		χ^2^_PS_B_ (2) = 11.986, *p* = 0.003
CFI_PS_B_	1.000		0.884
RMSEA_PS_B_	0.000		0.175
**LEVEL-SPECIFIC APPROACH FOR LEVEL-1 MODEL (PS_W)**			
Test of exact fit	χ^2^_PS_W_ (1) = 0.676, *p* = 0.411	χ^2^_PS_W_ (2) = 256.812^a^	
CFI_PS_W_	1.000	0.959	
RMSEA_PS_W_	0.000	0.149	
**SEGREGATING PROCEDURE FOR LEVEL-2 MODEL (YB_B)**			
Test of exact fit	χ^2^_YB_B_ (1) = 2.742, *p* = 0.098		χ^2^_YB_B_ (2) = 26.120^a^
CFI_YB_B_	0.994		0.921
RMSEA_YB_B_	0.103		0.272
**SEGREGATING PROCEDURE FOR LEVEL-1 MODEL (YB_W)**			
Test of exact fit	χ^2^_YB_W_ (1) = 0.677, *p* = 0.411	χ^2^_YB_W_ (2) = 256.748^a^	
CFI_YB_W_	1.000	0.959	
RMSEA_YB_W_	0.000	0.149	

a *p < 0.001. The degrees of freedom for test of exact fit are shown in parentheses. Standard, model fit evaluation for the entire model using the standard approach (i.e., both level-1 and level-2 models are evaluated simultaneously); PS_B, fit evaluation for level-2 model obtained using the level-specific approach; PS_W, fit evaluation for level-1 model obtained using the level-specific approach; YB_B, fit evaluation for level-2 model obtained using the segregating procedure; YB_W, fit evaluation for level-1 model obtained using the segregating procedure*.

**Table 2:** **Estimated two-level path model of mathematics motivation, gender, and achievement (Model_C_)**.

**Structural path**	**Multilevel model**	**Segregated model**
**LEVEL 1**		
MConf → MAch	−2.929 (0.181)^*^	−2.928 (0.181)^*^
MUtil → MAch	−0.909 (0.205)^*^	−0.909 (0.205)^*^
MInt → MAch	−0.558 (0.169)^*^	−0.559 (0.169)^*^
Female → MConf	0.165 (0.019)^*^	0.165 (0.019)^*^
Female → MInt	0.052 (0.020)^*^	0.052 (0.020)^*^
Female → MAch	0.957 (0.222)^*^	0.957 (0.222)^*^
**LEVEL 2**		
MConf → MAch	−36.850 (4.967)^*^	−36.779 (2.536)^*^
MUtil → MAch	7.460 (11.228)	7.363 (4.716)
MInt → MAch	13.455 (7.025)^†^	13.421 (3.235)^*^
Female → MConf	0.056 (0.065)	0.050 (0.046)
Female → MInt	0.069 (0.060)	0.062 (0.036)
Female → MAch	6.844 (2.001)^*^	6.793 (1.344)^*^

Multilevel model, maximum likelihood (ML) estimates obtained by multilevel structural equation modeling. Segregated model, ML estimates obtained from separate single-level path models using Yuan and Bentler's segregating procedure. Standard errors are shown in parentheses.

* p < 0.05;

† *p = 0.055*.

For Model_MW_, in which the structural relationship between MConf_W_ and MAch_W_ was constrained to be zero, all three approaches detected the lack of fit due to the constraint. The limitation of the standard approach is that the fit statistics are likely to be dominated by the level-1 model because of larger sample size. In this example, the effective sample size for the level-1 model was 5764, which is much larger than the level-2 effective samples size 164. The limitation of the standard approach was less severe for the level-1 model. The level-2 model was the same for Model_C_ and Model_MW_, and the fit statistics for the level-2 model (PS_B and YB_B) were identical.

Model_MB_, in which the structural relationship between Female_B_ and MAch_B_ is constrained to be zero, demonstrates the limitation of the standard approach and the advantage of the alternative approaches. In the standard approach, both the CFI and RMSEA indicated good model fit. However, the level-specific (PS_B) and segregating (YB_B) approaches produced the fit indices that indicate poor model fit. In Model_C_, the proportion of females was significantly related to the aggregated level of mathematic achievement at level 2. The estimated difference in mathematics achievement was 6.844 in terms of the Singapore national math Rasch score (i.e., 0.684 standard deviation) between schools with no female students (Female_B_ = 0) and schools with only female students (Female_B_ = 1). Alternatively the estimate can be interpreted that the estimated difference in Singapore national math Rasch score was 3.422 (i.e., 0.342 standard deviation) between schools with 25% female students and schools with 75% female students. If the researcher relied on the standard approach to evaluating model fit, she would have incorrectly concluded that the proportion of females is not related to the aggregated level of mathematics achievement. The level-1 model was the same for Model_C_ and Model_MB_, and the fit statistics for the level-1 model (PS_W and YB_W) were identical.

## Concluding remarks

Establishing a well-fitting model is an important and necessary step in applications of MSEM. A conventional practice has been that the model fit is evaluated using the fit statistics parallel to those developed for single-level SEM. Recently, a number of studies have pointed out limitations of the standard approach and have proposed alternative methods to overcome the limitations. This article reviewed two alternative approaches, both of which provide statistical methods to evaluate the model at each level separately. Simulation studies have shown that both approaches perform well in detecting lack of fit at both levels, whereas the standard approach failed to detect lack of fit in the level-2 model. As mentioned earlier, MSEM usually address two issues: (a) assessing the goodness of fit of the hypothesized model to the data, and (b) estimating and testing individual parameters in the hypothesized model. It is recommended that one or both of the alternative approaches are used to address (a). It is not recommended that the alternative approaches are used to address (b). Once a well-fitting model is selected, the parameter estimates and the standard errors in the selected model should be obtained using an estimation method which finds a solution for the whole set of parameters in the entire multilevel model (i.e., estimation method developed for MSEM). The ability of the level-specific and segregating approaches to assess model fit separately at each level provides researchers with valuable information in the evaluation of MSEM models.

### Conflict of interest statement

The author declares that the research was conducted in the absence of any commercial or financial relationships that could be construed as a potential conflict of interest.

## Footnotes

^1^The baseline model should be nested within the hypothesized model, i.e., the baseline model can be specified by adding constraints in the hypothesized model. In some applications, the independence model is not appropriate as a baseline model to compute CFI because the independence model is not nested within the hypothesized model. In this case, an alternative baseline model must be used (Widaman and Thompson, 2003).

^2^As an example, for the set of 5 variables, the SAS program failed to run due to insufficient memory for a sample size of 6597 clustered in 281 schools when executed in Intel(R) Core 2 Quad CPU @ 2.83 GHz, 3.23 GB of RAM.

^3^The mean structure is ignored in the segregating procedure. The mean structure in the example model was saturated, and therefore ignoring the mean structure did not affect the overall model fit.

^4^In the segregating procedure, the RMSEA_YB_B_ for the level-2 model was 0.103 (greater than the conventional rule of thumb < 0.08 for adequate fit). But the 90% confidence interval for the RMSEA_YB_B_ was [0.000, 0.258], and the *p* value for *H*_0_: RMSEA_YB_B_ ≤ 0.05 was 0.166. Also examination of the residual covariance matrices supported good model fit (the largest normalized residual was small than 2). Note that the level-2 sample size was 164.

^5^Due to limited space, only the estimates and standard errors of structural paths are presented. The estimates for other parameters are available upon request to the author.
